# Phase III randomized trial comparing moderate-dose cisplatin to combined cisplatin and carboplatin in addition to mitomycin and ifosfamide in patients with stage IV non-small-cell lung cancer

**DOI:** 10.1054/bjoc.2000.1413

**Published:** 2000-11

**Authors:** J-P Sculier, J-J Lafitte, M Paesmans, J Thiriaux, C G Alexopoulos, J Baumöhl, J Schmerber, G Koumakis, M C Florin, C Zacharias, T Berghmans, P Mommen, V Ninane, J Klastersky

**Affiliations:** Service de Médecine, For the European Lung Cancer Working Party, Institut Jules Bordet, 1 rue Héger-Bordet, Brussels, B-1000, Belgium

**Keywords:** non-small cell lung cancer, randomized trial, cisplatin, stage IV

## Abstract

A phase III randomized trial was conducted in patients with metastatic NSCLC, to determine if, in association with mitomycin (6 mg m^–2^) and ifosfamide (3 g m^–2^), the combination of moderate dosages of cisplatin (60 mg m^–2^) and carboplatin (200 mg m^–2^) – CarboMIP regimen – improved survival in comparison with cisplatin (50 mg m^–2^) alone – MIP regimen. A total of 305 patients with no prior chemotherapy were randomized, including 297 patients assessable for survival (147 in the MIP arm and 150 in the CarboMIP arm) and 268 patients assessable for response to chemotherapy. All but eight (with malignant pleural effusion) had stage IV disease. There was a 27% (95% CI, 19–34) objective response (OR) rate to MIP (25% of the eligible patients) and a 33% (95% CI, 24–41) OR rate to CarboMIP (29% of the eligible patients). This difference was not statistically significant (*P* = 0.34). Duration of response was not significantly different between both arms. There was also no difference (*P* = 0.67) in survival: median survival times were 28 weeks (95% Cl, 24–32) for MIP and 32 weeks (95% Cl, 26–35) for CarboMIP, with respectively 1-year survival rates of 24% and 23% and 2-year survival rates of 5% and 2%. The main toxicities consisted in emesis, alopecia, leucopenia and thrombocytopenia, that were, except alopecia, significantly more severe in the CarboMIP arm. Our trial failed to demonstrate a significant improvement in response or survival when patients with metastatic NSCLC were treated, in addition to ifosfamide and mitomycin, by combination of moderate dosages of cisplatin and carboplatin instead of moderate dosage of cisplatin alone. The results support the use of a moderate dose (50 mg m^–2^) of cisplatin in combination with ifosfamide and mitomycin for the chemotherapy of this disease. © 2000 Cancer Research Campaign


*British Journal of Cancer* (2000) **83**(9), 1128–1135
© 2000 Cancer Research Campaign
doi: 10.1054/ bjoc.2000.1413, available online at http://www.idealibrary.com on IDEAL®

### Phase III randomized trial comparing moderate-dose cisplatin to combined cisplatin and carboplatin in addition to mitomycin and ifosfamide in patients with stage IV non-small-cell lung cancer

**J-P Sculier[1], J-J Lafitte, M Paesmans, J Thiriaux, CG Alexopoulos, J Baumöhl, J Schmerber, G Koumakis, MC Florin, C Zacharias, T Berghmans, P Mommen, V Ninane and J Klastersky**

For the European Lung Cancer Working Party; [1]Service de Médecine, Institut Jules Bordet, 1 rue Héger-Bordet, B-1000 Brussels, Belgium

**Summary** A phase III randomized trial was conducted in patients with metastatic NSCLC, to determine if, in association with mitomycin (6 mg m$^{-2}$) and ifosfamide (3 g m$^{-2}$), the combination of moderate dosages of cisplatin (60 mg m$^{-2}$) and carboplatin (200 mg m$^{-2}$) – CarboMIP regimen – improved survival in comparison with cisplatin (50 mg m$^{-2}$) alone – MIP regimen. A total of 305 patients with no prior chemotherapy were randomized, including 297 patients assessable for survival (147 in the MIP arm and 150 in the CarboMIP arm) and 268 patients assessable for response to chemotherapy. All but eight (with malignant pleural effusion) had stage IV disease. There was a 27% (95% CI, 19–34) objective response (OR) rate to MIP (25% of the eligible patients) and a 33% (95% CI, 24–41) OR rate to CarboMIP (29% of the eligible patients). This difference was not statistically significant ($P$ = 0.34). Duration of response was not significantly different between both arms. There was also no difference ($P$ = 0.67) in survival: median survival times were 28 weeks (95% CI, 24–32) for MIP and 32 weeks (95% CI, 26–35) for CarboMIP, with respectively 1-year survival rates of 24% and 23% and 2-year survival rates of 5% and 2%. The main toxicities consisted in emesis, alopecia, leucopenia and thrombocytopenia, that were, except alopecia, significantly more severe in the CarboMIP arm. Our trial failed to demonstrate a significant improvement in response or survival when patients with metastatic NSCLC were treated, in addition to ifosfamide and mitomycin, by combination of moderate dosages of cisplatin and carboplatin instead of moderate dosage of cisplatin alone. The results support the use of a moderate dose (50 mg m$^{-2}$) of cisplatin in combination with ifosfamide and mitomycin for the chemotherapy of this disease. © 2000 Cancer Research Campaign

**Keywords:** non-small cell lung cancer; randomized trial; cisplatin; stage IV

The administration of chemotherapy has allowed improvement of survival in comparison to best supportive care alone (Grilli et al, 1993; Souquet et al, 1993; Marino et al, 1994; Sculier et al, 1999*a*) in metastatic non-small cell lung cancer (NSCLC). Moreover, it was also found to be associated with a better quality of life (Thongprasert et al, 1999) and a reduced economic cost (Jaakkimainen et al, 1990). The classical active cytostatic agents for NSCLC are cisplatin, mitomycin, vindesine, vinblastine and ifosfamide (Donnadlieu et al, 1991). New active drugs recently introduced are paclitaxel, docetaxel, gemcitabine and vinorelbine (Meert et al, 1999).

Cisplatin is considered so far to be the most important drug. A series of randomized trials (Fuks et al, 1983; Elliott et al, 1984; Robert et al, 1984; Einhorn et al, 1986; Luedke et al, 1990; Rosso et al, 1990; Depierre et al, 1994; Le Chevalier et al, 1994) have shown that the addition of cisplatin to a combination of other cytostatic agents improves the objective response rates in the majority of the studies and prolongs survival with a statistically significant comparison test in half of them. The dosage of cisplatin has been investigated in four randomized trials (Gralla et al, 1981; Klastersky et al, 1986; Shinkai et al, 1986; Gandara et al, 1993). Two of them included very small numbers of patients. When combined with etoposide or vindesine, there seems to be no advantage in terms of response or survival to administer high cisplatin doses (120 mg m$^{-2}$) in comparison to moderate doses (60 or 80 mg m$^{-2}$).

A major issue with the higher doses of cisplatin is delayed toxicity: our Group (see Appendix) has shown (Sculier et al, 1994*a*) that the risk of developing a WHO Grade II neurological (polyneuropathy), auditory (hypoacousia) or renal (renal failure) toxicity, after six courses of high-dose cisplatin (120 mg m$^{-2}$), is about 25%. This toxicity precludes further therapy and probably reduces the impact of cisplatin therapy on survival. Replacement of cisplatin by its better-tolerated analogue, carboplatin, has been disappointing because it induces less favourable results, at least in terms of response rate (Klastersky et al, 1990). But, if moderate dosages of carboplatin (200 mg m$^{-2}$) and cisplatin (60 mg m$^{-2}$) are combined (Sculier et al, 1994*a*), the same activity as high doses of cisplatin (120 mg m$^{-2}$) is maintained in terms of response and survival with a reduced risk of long-term renal, neurological or auditory toxicity (6% at six courses of therapy). In this regimen, 100 mg m$^{-2}$ of carboplatin is considered equivalent to 30 mg m$^{-2}$ of cisplatin. As previously discussed (Sculier et al, 1994*a*), this equivalence is extrapolated from experimental data, no clinical data being available.

The primary purpose of the present trial is to determine if, in patients with stage IV NSCLC, the combination of moderate

*Received 29 February 2000*
*Revised 29 May 2000*
*Accepted 27 June 2000*

*Correspondence to*: J-P Sculier dosages of cisplatin and carboplatin improves survival in comparison with cisplatin alone in association with mitomycin and ifosfamide as used in the Cullen regimen (Cullen et al, 1988).

### PATIENTS AND METHODS

### Selection criteria

Patients with histologically proven NSCLC had to fulfil all the following criteria to enter this study: inoperable stage IV or stage IIIB with malignant pleural effusion; presence of a measurable or assessable lesion; no prior history of malignancy except non-melanoma skin cancer, in situ carcinoma of the cervix or 'cured' malignant tumour (more than 5-year disease-free survival); no prior chemotherapy; Karnofsky performance status (PS) ≥ 60; good renal (serum creatinine level ≤ 1.5 mg $dl^{-1}$ and/or creatinine clearance ≥ 60 ml $min^{-1}$), hepatic (serum bilirubin level ≤ 1.5 mg $dl^{-1}$) and haematological (WBC count ≥ 4000 $mm^{-3}$ and platelet count ≥ 100 000 $mm^{-3}$) functions; no recent (less than 3 months before the date of treatment) myocardial infarction and no active congestive heart failure or cardiac arrhythmia requiring medical treatment; no uncontrolled infectious disease; no other serious medical or psychological factors which may prevent adherence to the treatment schedule. Patients had to be accessible for follow-up and had to provide informed consent. Protocol had to have been approved by the local ethical committee of the investigator.

### Treatment

Eligible patients were randomized between two arms: the CarboMIP regimen consisting of moderate doses of a combination of cisplatin (CDDP) (60 mg $m^{-2}$) and carboplatin (CBDCA) (200 mg $m^{-2}$ plus mitomycin C (6 mg $m^{-2}$) and ifosfamide (3 g $m^{-2}$) and the MIP regimen consisting of moderate doses of cisplatin (50 mg $m^{-2}$) plus mitomycin C and ifosfamide (same dosages as in the CarboMIP regimen).

All drugs were given i.v. on day 1 of the cycle. Mitomycin C was given as a bolus followed by ifosfamide infused over 3 h. Cisplatin (Platinol®) was administered over 1 h, 3 h after the end of the ifosfamide infusion. Mesna (Uromitexan®) (1 g $m^{-2}$ 3 h infusion together with ifosfamide (Holoxan®), followed by 500 mg $m^{-2}$ bolus at 4 and 8 h) was provided to avoid urotoxicity, according to the initial Cullen publication (Cullen et al, 1988). Carboplatin (Paraplatin®) was administered in ready-to-use solution over 30 min just before cisplatin infusion. Platinum derivatives administration was followed by i.v. infusion of 2000 ml NaCl 0.9% with 3 g KCl over 16 h. To control emesis, the administration of a 5-HT3 antagonist (granisetron) was recommended in combination with dexamethasone and/or lorazepam.

Courses were given every 3 weeks. Tumour response was assessed after three full courses. In case of stable disease or progression, the treatment was discontinued. Responding patients were given additional courses until best response, progression (PG) or major toxicity.

Courses were repeated as soon as haematological (WBC > 4000 $mm^{-3}$ and platelets > 100 000 $mm^{-3}$) and renal (serum creatinine < 1.5 mg $dl^{-1}$) functions had recovered. If delay between two courses was more than 5 weeks, patient went off-treatment. If WBC nadir was < 1000 $mm^{-3}$ and/or platelet nadir < 25 000 $mm^{-3}$, carboplatin, ifosfamide and mitomycin C dosage were reduced to 75% during the following courses. If serum creatinine peak increased between 1.5 and 3.0 mg $dl^{-1}$, cisplatin dosage was reduced to 50%. If it was > 3.0 mg $dl^{-1}$, cisplatin was stopped. In case of occurrence of hearing loss or > WHO grade II neurotoxicity, cisplatin was also stopped.

### Investigations

The initial work-up consisted of complete history and physical examination with weight, height and surface-area measurements; recording of performance status; fibreoptic bronchoscopy with biopsy; bone scintigraphy; liver and adrenals computed tomography (CT) scan or echography; brain CT scan; blood chemistries including complete blood-cell count, electrolytes, serum creatinine and liver function tests; EKG.

Blood chemistries, chest X-ray, and clinical examination were repeated before each course. Restaging with all tests performed during the initial work-up was repeated after the first three courses and every three courses if treatment was continued.

After treatment completion, patients were followed up every 2 months for the first 6 months and then every 3 months, with clinical examination, chest X-rays and biological tests.

### Criteria of evaluation

Patients were evaluated for response after completion of three courses of chemotherapy. Responses were evaluated by at least three independent observers during regular meetings of the Group. Complete remission (CR) was defined as the disappearance of all signs of disease, for at least 4 weeks. Partial response (PR), in measurable disease, consisted of 50% or greater decrease of the total tumour load as established by two observations not less than 4 weeks apart and without the appearance of new lesions or progression of any lesion. The tumour load was estimated as the tumour area calculated by the multiplication of the longest diameter by the greatest perpendicular diameter. In assessable disease, PR was defined as an estimated decrease in tumour size of 50% or more. PG was considered to be an increase of greater than 25% in one or more measurable or assessable lesions or the appearance of a new lesion. All other circumstances were classified as no change (NC). Patients with early death (ED) due to disease progression before evaluation, those with toxic death due to chemotherapy, or those with early chemotherapy stoppage for toxicity, were considered as treatment failures.

Duration of response was calculated from the day of registration until the date of the first observation of PG. Survival was dated from the day of registration. WHO criteria were used to assess toxicity.

### Primary endpoint and sample-size determination

The primary objective of the trial was to compare the survival distributions obtained with the MIP and CarboMIP regimens. The sample-size evaluation was based on this endpoint. On the basis of the Group's previous experience, for the MIP regimen we expected a 1-year survival rate of 20%. It was judged that an improvement of this rate from 20–35% would be clinically meaningful. With these assumptions and using a two-sided logrank test for the primary comparison of the two arms of the study ($\alpha$ = 5%, $\beta$ = 20%), 123 eligible patients needed to be randomized in each

© 2000 Cancer Research Campaign arm of the study and the analysis could be performed after 89 deaths in each group. As there were no formal data on the toxicity of the CarboMIP combination, an interim analysis on the tolerance of the regimen was performed after evaluation of the first 20 randomized patients. No interim analysis for survival was planned or performed.

### Randomization procedure

Randomization was centrally performed using the minimization technique and stratified according to centre, presence of brain metastases, existence of prior chest irradiation and Karnofsky performance status. Treatment allocation was obtained by calling the ELCWP data center.

### Statistical methodology

Survival curves were estimated by the method of Kaplan and Meier. The log-rank test was used to compare survival curves. *P*-values for testing differences between proportions were calculated with Chi-square tests or with Fisher's exact tests. A multivariate analysis for comparison adjustment taking into account prognostic factors was performed by fitting the data with a Cox model for duration of survival and a logistic regression model for objective response.

The evaluation of chemotherapy intensity was performed by calculating absolute and relative dose intensities. The absolute dose intensity (ADI) was defined as the ratio of the received dose per $m^{-2}$ body surface to the actual duration of treatment: it is expressed in mg $m^{-2}$ $week^{-1}$. Carboplatin was considered equivalent to cisplatin in the ratio 100/30. All the formulas have been previously published (Sculier et al, 1996). Comparisons of the distributions of the dose intensity variables between regimens have been done by using the Mann–Whitney test.

The influence of the modality of prescription of carboplatin according to the body surface or to the carboplatin clearance related to the renal function was evaluated according to the Calvert AUC-based equation. As creatinine clearance was not systematically available, glomerular filtration rate (GFR) was evaluated according to the Cockcroft formula, from serum creatinine that was measured before each new cycle of chemotherapy. The method reported by Chatelut was also used to calculate the carboplatin clearance according to weight, age, sex and serum creatinine. All the formulas and references have been reported in a prior publication (Sculier et al, 1999*b*).

### RESULTS

### Patient population and characteristics

A total of 305 patients were randomized between October 1995 and March 1998. Eight (2.6%) were ineligible for the study (five on the MIP arm and three on the CarboMIP arm) for the following reasons: no assessable lesion (*n* = 5), presence of a second active cancer (*n* = 2), patient inaccessible to follow-up (*n* = 1). Among the 297 patients assessable for survival, 29 (9.8%) were not assessable for response (11 on the MIP arm and 18 on the CarboMIP arm), because of loss of follow up (*n* = 3), incomplete assessment work-up (*n* = 1), major protocol violation (*n* = 3), early death not reported as due to disease or treatment toxicity (*n* = 16, including six sudden deaths), too long a delay between two courses of therapy (*n* = 1), chemotherapy discontinuation for intercurrent disease (*n* = 2), patient's refusal of further treatment (*n* = 1), death before treatment (*n* = 2). Thus, 268 patients were assessable for response.

**Table 1** Patient characteristics

| | Treatment arm | |
|---|---|---|
| | **MIP** | **CarboMIP** |
| Eligible patients (*n*) | 147 | 150 |
| Sex | | |
| male | 116 | 130 |
| Female | 31 | 20 |
| Age in years | | |
| Median | 61 | 62 |
| Range | 32–78 | 34–75 |
| Karnofsky PS | | |
| ≤ 70 | 48 | 50 |
| ≥ 80 | 99 | 100 |
| Type of lesions | | |
| Assessable | 70 | 77 |
| Measurable | 77 | 73 |
| Loss of body weight | | |
| < 5% | 84 | 82 |
| ≥ 5% | 52 | 60 |
| Unknown | 11 | 8 |
| Histology | | |
| Squamous cell carcinoma | 42 | 57 |
| Adenocarcinoma | 75 | 66 |
| Other non-small-cell | 30 | 27 |
| Disease extent | | |
| Stage IIIB | 4 | 4 |
| Stage IV | 143 | 146 |
| Brain metastases (*n*) | 42 | 37 |
| Prior chest irradiation (*n*) | 11 | 7 |
| Prior surgery (*n*) | 13 | 18 |

Characteristics of the eligible patients are listed in Table 1. Both arms were well balanced for the main patients characteristics. There were 147 patients on the MIP arm and 150 on the CarboMIP arm. The majority of the patients were male (83%) and had a good Karnofsky PS (≥ 80 in 67%). Only eight (3%) presented with stage IIIB disease and malignant pleural effusion and 18 (6%) had undergone prior chest irradiation. Histology was adenocarcinoma in 47.5% and squamous cell carcinoma in 33.3%. Twenty seven percent of the patients had brain metastases.

The median follow-up duration was 96 weeks (range, 1–168). At the time of the present analysis, 261 patients were dead, 36 were alive, and two had been lost to follow-up evaluation.

### Tumour response

As shown in Table 2, there was a 27% (95% CI, 19–34) OR rate to MIP (25% of eligible patients; 95% CI, 17–32) and a 33% (95% CI, 24–41) OR rate to CarboMIP (29% of the eligible patients; 95% CI, 21–36). This difference was not statistically significant (*P* = 0.34). In none of subgroups of patients with a similar characteristic (age, sex, weight-loss, PS, type of lesion, histology, presence of brain metastases, prior surgery or radiotherapy) was there a significant difference in response rates between the two arms.

By univariate analysis performed on all eligible patients (Table 3), six variables were found to be significant prognostic factors for a higher response rate: squamous cell carcinoma, absence of brain metastases, normal serum alkaline phosphatases, no prior local treatment, a lower serum creatinine level and a Karnofsky PS > 70.

 © 2000 Cancer Research Campaign

**Table 2** Evaluation of response

| Results | Treatment arm | |
|---|---|---|
| | MIP | CarboMIP |
| Patients (*n*) | 147 | 150 |
| Not assessable for response (*n*) | 11 | 18 |
| Assessable for response (*n*) | 136 | 132 |
| PR (*n*) | 36 | 43 |
| OR rate (%) | 27 | 33 |
| NC (*n*) | 45 | 39 |
| PG (*n*) | 43 | 30 |
| ED due to cancer (*n*) | 7 | 4 |
| Toxic death (*n*) | 2 | 5 |
| Removal because of excessive toxicity (*n*) | 3 | 10 |
| Death by tumoural necrosis (*n*) | 0 | 1 |

PR = partial response; OR = objective response; NC = no change; PG = progression; ED = early death

A multivariate analysis with data fitted using logistic regression model selected with a backward stepwise method was performed. The potential covariates were all of the aforementioned significant ones plus neutrophil count in a subset of 270 assessable patients who had all data available. Significant independent factors identified were histology ($P$ = 0.001), prior local treatment ($P$ = 0.01), brain metastases ($P$ = 0.01), serum alkaline phosphatases ($P$ = 0.02) and Karnofsky PS ($P$ = 0.05). Increased odds ratios for a higher OR rate were 1.90 (95% CI, 1.00–3.69) for PS > 70 and 2.08 (95% CI, 1.09–3.96) for increased alkaline phosphatases. Decreased odds ratios for a lower OR rate were 0.33 (95% CI, 0.17–0.62) for adenocarcinoma and 0.35 (95% CI, 0.16–0.81) for other NSCLC subtypes, 0.41 (95% CI, 0.19–0.86) for the presence of brain metastases and 0.22 (95% CI, 0.06–0.77) for prior local treatment.

### Duration of response

Duration of response was not significantly different between the arms, with a median of 23 weeks (95% CI, 21–29) for MIP and 30 weeks (95% CI, 24–36) for CarboMIP. The progression-free survival was also not significantly different between both treatments.

### Survival

There was no difference ($P$ = 0.67) in survival between the study arms (Figure 1): median survival times were 28 weeks (95% CI, 24–32) for MIP and 32 weeks (95% CI, 26–35) for CarboMIP with respectively 1-year survival rates of 24% and 23% and 2-year survival rates of 5% and 2%. At time of analysis, 134 deaths were observed in the MIP arm and 129 in the CarboMIP arm. In no subgroup defined by a clinical characteristic (age, sex, PS, weight-loss, type of lesions, histology, brain metastases, prior local therapy) was a statistically significant difference found for survival, according to regimen.

Some variables were identified by univariate analysis (Table 3) as significantly associated with improved survival: female sex, PS ≥ 80, body weight-loss ≤ 5%, prior local therapy, normal white blood cells, normal neutrophil count, normal haemoglobin level, normal alkaline phosphatases, normal LDH level and serum creatinine ≥ 1 mg $dl^{-1}$. A Cox model, using all variables with a $P$-value < 0.3 in univariate analysis (except LDH and weight-loss), performed in 270 patients for which the data were available, selected as significant independent factors for survival: sex (HR 0.62 in favour of female; 95% CI, 0.43–0.90; $P$ = 0.008), PS (HR 0.60 in favour of PS > 70; 95% CI, 0.45–0.78; $P$ < 0.001), prior local therapy (HR 0.57 in favour of prior treatment; 95% CI, 0.37–0.88; $P$ = 0.006) and neutrophils (HR 1.73 in favour of normal count; 95% CI, 1.32–2.28; $P$ < 0.001). When performed on a restricted set of 211 patients for which LDH and weight-loss data were available, the same factors were found as significant, except sex that was not anymore selected and histology that was an independent predictor in favour of adenocarcinoma.

**Table 3** Univariate prognostic factors analysis

| Factor | Response (%) | *P* | Median survival (weeks) | *P* |
|---|---|---|---|---|
| Treatment | | | | |
| MIP | 25 | | 28 | |
| CarboMIP | 29 | 0.34 | 32 | 0.67 |
| Age | | | | |
| < 60 years | 24 | | 30 | |
| ≥ 60 years | 28 | 0.53 | 29 | 0.87 |
| Sex | | | | |
| Male | 27 | | 29 | |
| Female | 26 | 0.98 | 37 | 0.02 |
| Weight-loss | | | | |
| ≤ 5% | 30 | | 35 | |
| > 5% | 24 | 0.39 | 24 | 0.002 |
| Karnofsky PS | | | | |
| ≤ 70 | 17 | | 20 | |
| ≥ 80 | 31 | 0.02 | 35 | < 0.001 |
| Lesion type | | | | |
| Assessable | 26 | | 32 | |
| Measurable | 27 | 0.87 | 28 | 0.49 |
| Histology | | | | |
| Squamous cell carcinoma | 41 | | 30 | |
| Adenocarcinoma | 18 | | 33 | |
| Other types of NSCLC | 23 | < 0.001 | 24 | 0.09 |
| Brain metastases | | | | |
| Absent | 31 | | 32 | |
| Present | 14 | 0.005 | 24 | 0.06 |
| Prior surgery or radiotherapy | | | | |
| No | 29 | | 29 | |
| Yes | 8 | 0.01 | 50 | 0.003 |
| WBC (per $mm^3$) | | | | |
| ≤ 10 000 | 29 | | 37 | |
| > 10 000 | 24 | 0.39 | 23 | < 0.001 |
| Neutrophils (per $mm^3$) | | | | |
| ≤ 7500 | 30 | | 37 | |
| > 7500 | 22 | 0.14 | 18 | < 0.001 |
| Platelets (per $mm^3$) | | | | |
| ≤ 440 000 | 27 | | 31 | |
| > 440 000 | 26 | 1 | 26 | 0.19 |
| Haemoglobinaemia (g $dl^{-1}$) | | | | |
| 12–18 | 29 | | 33 | |
| < 12 or > 18 | 22 | 0.31 | 21 | 0.002 |
| Alkaline phosphatases (mU $ml^{-1}$) | | | | |
| ≤ 110 | 17 | | 35 | |
| > 110 | 33 | 0.006 | 28 | 0.04 |
| LDH (mU $ml^{-1}$) | | | | |
| ≤ 200 | 23 | | 37 | |
| > 200 | 29 | 0.40 | 28 | 0.02 |
| Serum creatinine (mg $dl^{-1}$) | | | | |
| ≤ 1 | 25 | | 28 | |
| > 1 | 35 | 0.25 | 41 | 0.03 |

*© 2000 Cancer Research Campaign* 

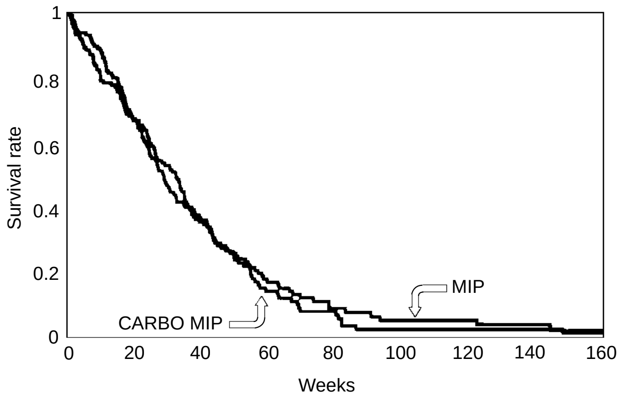

**Figure 1** Survival according to treatment

### Toxicity

As summarized in Table 4, the main toxicities consisted in emesis, alopecia, leucopenia and thrombocytopenia. Except alopecia, those events were significantly more severe on the CarboMIP arm. Chronic auditory, renal and neurological toxicity were infrequent and the risk of developing at least one grade II–IV of one of these toxicities was not significantly different between the two study arms (6% vs 8% after three courses and 9% vs 11% after six courses, respectively, for MIP and CarboMIP). There were seven toxic deaths: two on the MIP arm (all febrile neutropenia) and five on the CarboMIP arm (three febrile neutropenia, one acute renal failure and one cerebral stroke).

### Dose intensity

Median (range) cumulative doses of drugs per patient, for the MIP and CarboMIP arms respectively, were: 151 (49–313) and 361 (0–733) mg $m^{-2}$ for platinum derivatives (in cisplatinum-equivalent), 18 (5.6–37.5) and 18.2 (0–36.9) mg $m^{-2}$ for mitomycin ($P$ = 0.16) and 9.0 (2.8–18.9) and 9.1 (0–22.2) g $m^{-2}$ for ifosfamide ($P$ = 0.30). Median absolute dose-intensity was, respectively for patients treated respectively by MIP and CarboMIP, 15.9 (9.1–19.9) and 34.4 (0–41.8) mg $m^{-2}$ $wk^{-1}$ for platinum derivatives, 1.9 (0.9–2.1) and 1.7 (0–2.1) mg $m^{-2}$ $wk^{-1}$ for mitomycin ($P$ < 0.001) and 0.95 (0.33–1.13) and 0.88 (0–1.35) g $m^{-2}$ $wk^{-1}$ for ifosfamide ($P$ < 0.001).

The potential influence of the modality of prescription of carboplatin was retrospectively evaluated by calculating the carboplatin dosing according to the Calvert AUC-based equation and the glomerular filtration rate (GFR) by the Cockcroft and Chatelut methods. There was a good linear relationship between the results obtained by both methods. For the first course of chemotherapy, the median AUC given was retrospectively calculated to be 3.6 for the two methods. Nineteen per cent of the patients had received an AUC < 3 using the Cockcroft equation compared with 20% using the Chatelut method. Haematological toxicity did not appear to be significantly associated with the AUC administered, except for course 1 and grade III/IV leucopenia (15% toxicity if AUC < 3 vs 44% if AUC ≥ 3; $P$ = 0.01).

**Table 4** Main toxicities for the first three courses per patient per arm

| Toxicity | Treatment arm MIP | Treatment arm CarboMIP | *P* |
|---|---|---|---|
| Acute (% grade III–IV) | | | |
| Emesis | 4 | 12 | 0.04 |
| Alopecia | 17 | 26 | 0.11 |
| Leucopenia | 35 | 65 | < 0.001 |
| Infection | 9 | 9 | 1 |
| Thrombocytopenia | 14 | 54 | < 0.001 |
| Bleeding | 0 | 1 | 0.24 |
| Chronic (% grade II–IV) | | | |
| Auditory | 0 | 0 | 1 |
| Renal | 0 | 5 | 0.008 |
| Neurological | 7 | 5 | 0.40 |
| At least one of these | 6 | 8 | 0.68 |

### DISCUSSION

The principal finding of this trial, conducted in metastatic NSCLC, was the lack of improvement in terms of response rate and survival when a combination of moderate dosage cisplatin (60 mg $m^{-2}$) and carboplatin (200 mg $m^{-2}$) was substituted for moderate dosage cisplatin (50 mg $m^{-2}$) in a mitomycin–ifosfamide chemotherapy regimen. Our Group confirms, in some ways, the data reported in one of its previous trials (Klastersky et al, 1986) showing no significant change of response rate and survival when, in an etoposide containing regimen, 120 mg $m^{-2}$ cisplatin was compared to 60 mg $m^{-2}$ cisplatin in advanced NSCLC. In fact, none of the randomized trials testing the role of the dosage of cisplatin have so far reported significant differences (Gralla et al, 1984, Klastersky et al, 1986; Shinkai et al, 1986; Gandara et al, 1993). The only argument for a high dosage is based on a meta-analysis, conducted in the early nineties (Donnadieu et al, 1991), where we compared regimens with a ‘low’ dose (≤ 70 mg $m^{-2}$) to those with a ‘high’ dose (≥ 100 mg $m^{-2}$). Response rate was significantly higher ($P$ = 0.005) with high-dose regimens (34% or 278/819 patients) than with low-dose regimens (28% or 699/2497). In the present trial, we observed a similar difference in response rate (27 vs 33%); however, to detect such a theoretical difference ($\alpha$ = 5%, $\beta$ = 20%), a much larger accrual of patients would have been required (about 1000 per arm). Nevertheless, response rate is much less important than survival and as the survival curves were strictly superimposed, it is very unlikely that a benefit for the patient has been missed.

We have not used high-dose cisplatin for the ‘high-dose’ arm, but a combination of moderate dosages of cisplatin (60 mg $m^{-2}$) and carboplatin (200 mg $m^{-2}$). This approach is based on the findings of our two recent studies performed in metastatic NSCLC (Sculier et al, 1994*a*; 1998) showing that regimens combining moderate-dose cisplatin and carboplatin are as active as high-dose cisplatin alone, but with significantly less chronic toxicity. The present study confirms the low rate of occurrence of renal failure, ototoxicity or polyneuropathy with this platinum derivatives combination.

As the addition of carboplatin to a moderate dose of cisplatin fails to produce a significant survival advantage, a further question would be to determine if cisplatin might be replaced by carboplatin alone (given at 200 mg $m^{-2}$ or AUC 3–4). The potential advantage of that substitution would be easier chemotherapy administration but at the probable cost of greater haematological

© 2000 Cancer Research Campaign toxicity. A trial testing this question would in fact be the counterpart at a lower dosage level of another study of our Group (Klastersky et al, 1990), that compared both drugs at higher dosages in combination with etoposide, with a better response rate for cisplatin but without significant survival difference.

In our previous trial (Sculier et al, 1998), a retrospective analysis has shown (Sculier et al, 1999*b*) that the therapeutic index was improved when the carboplatin dosage was calculated according to the AUC, because of significantly lower haematological toxicity. The data of the present trial that includes a much smaller number of patients assessable for that question are nevertheless consistent with the above-mentioned analysis, a greater rate of leucopenia being observed with higher AUC. This observation has led our Group to prescribe, for further trials, carboplatin using the Calvert formula.

The choice of drugs that we used was mainly based on the publication of Cullen et al (1988). We have shown in the above-mentioned previous trial (Sculier et al, 1998) that the addition of ifosfamide to moderate-dose cisplatin and carboplatin very significantly improves the tumour response rate. Despite the fact that there was no apparent effect on survival in the study, as it is usual in trials adding other active drugs except vinorelbine to platinum (Klastersky et al, 1989; Kawahara et al, 1991; Wozniak et al, 1998), ifosfamide can be considered as having a positive effect. The role of mitomycin C nevertheless might be more controversial because none of the eight randomized trials testing the addition of this drug to a cisplatin-based regimen has shown a significant survival advantage (Einhorn et al, 1986; Bonomi et al, 1989; Crino et al, 1990; Fukuoka et al, 1991; Shinkai et al, 1991; Weick et al, 1991; Mylonakis et al, 1992; Gandara et al, 1993). Only two trials have shown an improvement in the response rate and actually they could be criticized: in the first (Bonomi et al, 1989), a lower dose of cisplatin was used in the arm with mitomycin and in the second (Gandara et al, 1993), an unusually very high dosage of cisplatin was administered (200 mg $m^{-2}$). For all these reasons, it is very doubtful that mitomycin played a clinically significant role in our study and our Group has decided not to use this drug in further trials.

The analysis of dose-intensity might provide a potential explanation for the absence of survival difference between both arms. Indeed in the CarboMIP arm, there was a significant absolute dose-intensity reduction for ifosfamide and mitomycin, as a result of more frequent dose reductions and treatment delays due to increased haematological toxicity, probably related to the administration of carboplatin. The potential benefit of a higher dose-intensity in platinum derivatives might thus have been cancelled by the lower ones of the two other drugs in this arm. A similar observation was made in the trial where we tested the role of the addition of ifosfamide to the platinum derivatives combination (Sculier et al, 1998).

The prognostic factor analysis has provided some new data in comparison to our prior experience (Sculier et al, 1994*b*; 1998; Paesmans et al, 1995; 1997; Borges et al, 1996). Prior local treatment (surgery and/or chest irradiation) has been found to be an independent predictor of poor response to chemotherapy but it predicted better survival. A potential explanation might be cancer growing more slowly. This observation is new because to the best of our knowledge, it has never been reported before in multivariate analysis, even in our recently published trial performed on exactly the same type of patients (Paesmans and Sculier, 1998). Nevertheless, in this latter, chemotherapy regimen was a very significant prognostic factor for response, contrary to the present trial. The adenocarcinoma histological subtype was a highly significant predictor for poor response to chemotherapy, as we have also observed in our previous studies (Borges et al, 1996; Sculier et al, 1998). Patients with brain metastases had also less frequently an objective response. Although these characteristics have no significant impact on survival, their effect on response-rate might be an explanation for the discrepancies observed between series of patients with the same criteria of selection and treated with the same regimen, but with an accrual of patients with different characteristics. We found, as we had previously in older studies, a better prognosis for survival in female (Paesmans et al, 1995) and in patients with non-increased neutrophil counts (Paesmans et al, 1995; Sculier et al, 1998). This latter factor, although rarely investigated by other groups, is consistently present in all our analyses performed in lung cancer patients. It may be related to the absence of infection and/or of paraneoplastic production of hormones or cytokines.

Finally, new active drugs have recently become available for the treatment of NSCLC and it can be argued that the regimens that we have studied are today obsolete. In fact, the available randomized trials directly comparing regimens with new drugs (the ‘second generation’) to regimens with older drugs (the ‘first generation’) have so far not shown an advantage in terms of survival for the new drugs (Giaccone et al, 1998; Crino et al, 1999), although some benefits in terms of response rate or quality of life have been reported. Moreover, the MIP regimen is considered in Europe as one of the standard chemotherapies for NSCLC (Crino et al, 1990; Cullen et al, 1999).

In conclusion, our trial fails to demonstrate a significant improvement in response rate or survival when patients with metastatic NSCLC are treated, with the addition to ifosfamide and mitomycin, by combination of moderate dosages of cisplatin (60 mg $m^{-2}$) and carboplatin (200 mg $m^{-2}$) instead of moderate dosage cisplatin (50 mg $m^{-2}$) alone. The results support the use of a moderate dose of cisplatin for the chemotherapy of this disease. The purpose of our next trial will be to compare, in this type of patient, regimens with or without cisplatin.

### REFERENCES

Bonomi PD, Finkelstein DM, Ruckdeschel JC, Blum RH, Green MD, Mason B, Hahn R, Tormey DC, Harris J, Comis R and Glick J (1989) Combination chemotherapy versus single agents followed by combination chemotherapy in stage iv non-small-cell lung cancer: a study of the Eastern Cooperative Oncology Group. *J Clin Oncol* **7**: 1602–1613

Borges M, Sculier JP, Paesmans M, Richez M, Bureau G, Dabouis G, Lecomte J, Michel J, Van Cutsem O, Schmerber J, Giner V, Berchier MC, Sergysels R, Mommen P and Klastersky J (1996) Prognostic factors for response to chemotherapy containing platinum derivatives in patients with unresectable non-small cell lung cancer (NSCLC). *Lung Cancer* **16**: 21–33

Crino L, Scagliotti GV, Ricci S, De Marinis F, Rinaldi M, Gridelli C, Ceribelli A, Bianco R, Marangolo M, Di Constanzo F, Sassi M, Barni S, Ravaioli A, Adamo V, Portalone L, Cruciani G, Masotti A, Ferrara G, Gozzelino F and Tonato M (1999) Gemcitabine and cisplatin versus mitomycin, ifosfamide, and cisplatin in advanced non-small-cell lung cancer: A randomized phase III study of the Italian Lung Cancer Project. *J Clin Oncol* **17**: 3522–3530

Crino L, Tonato M, Darwish S, Meacci ML, Corgna E, Di Constanzo F, Buzzi F, Fornari G, Santi E, Ballatori E, Santucci C and Davis S (1990) A randomized trial of three cisplatin-containing regimens in advanced non-small-cell lung cancer (NSCLC): a study of the Umbrian Lung Cancer Group. *Cancer Chemother Pharmacol* **26**: 52–56

© 2000 Cancer Research Campaign 

Cullen MH, Billingham LJ, Woodroffe CM, Chetiyawardana AD, Gower NH, Joshi R, Ferry DR, Rudd RM, Spiro SG, Cook JE, Trask C, Bessell E, Connolly CK, Tobias J and Souhami RL (1999) Mitomycin, ifosfamide, and cisplatin in unresectable non-small-cell lung cancer: effects on survival and quality of life. *J Clin Oncol* **17**: 3188–3194

Cullen MH, Joshi R, Chetiyawardana AD and Woodroffe CM (1988) Mitomycin, ifosfamide and cisplatin in non-small cell lung cancer: treatment good enough to compare. *Br J Cancer* **58**: 359–361

Depierre A, Chastang CI, Quoix E, Lebeau B, Blanchon F, Paillot N, Lemarie E, Milleron B, Moro D, Clavier J, Herman D, Tuchais E, Jacoulet P, Brechot JM, Cordier JF, Salal-Celigny P, Badri N and Benseval M (1994) Vinorelbine versus vinorelbine plus cisplatin in advanced non-small-cell lung cancer: a randomized trial. *Ann Oncol* **5**: 37–42

Donnadieu N, Paesmans M and Sculier JP (1991) Chimiothérapie des cancers bronchiques non à petites cellules. Méta-analyse de la littérature en fonction de l'extension de la maladie. *Rev Mal Resp* **8**: 197–204

Einhorn LH, Loehrer PJ, Williams SD, Meyers S, Gabrys T, Nattan SR, Woodburn, Drasga R, Songer J, Fisher W, Stephens D and Hui S (1986) Random prospective study of vindesine versus vindesine plus high-dose cisplatin versus vindesine plus cisplatine plus mitomycin C in advanced non-small-cell lung cancer. *J Clin Oncol* **4**: 1037–1043

Elliott JA, Ahmedzai S, Hole D, Dorward AJ, Stevenson RD, Kaye SB, Banham SW, Stack BHR and Calman KC (1984) Vindesine and cisplatin combination chemotherapy compared with vindesine as a single agent in the management of non-small cell lung cancer: a randomized study. *Eur J Cancer Clin Oncol* **20**: 1025–1032

Fuks JZ, Aisner J, Van Echo DA, Schipper H, Levitt M, Ostrow S and Wiernik Ph (1983) Randomized study of cyclophosphamide, doxorubicin, and etoposide (VP16–213) with or without cisplatinum in non-small cell lung cancer. *J Clin Oncol* **1**: 295–301

Fukuoka M, Masuda N, Furuse K, Negoro S, Takada M, Matsui K, Takifuji N, Kudoh S, Kawahara M, Ogawara M, Kodama N, Kubota K, Yamamoto M and Kusunoki Y (1991) A randomized trial in inoperable non-small-cell lung cancer: vindesine and cisplatin versus mitomycin, vindesine, and cisplatin versus etoposide and cisplatin alternating with vindesine and mitomycin. *J Clin Oncol* **9**: 606–613

Gandara DR, Crowley J, Livingston RB, Perez EA, Taylor CW, Weiss G, Neefe JR, Hutchins LF, Roach RW, Grunberg SM, Braun TJ, Natale RB and Balcerzak P (1993) Evaluation of cisplatin intensity in metastatic non-small-cell lung cancer: a phase III study of the Southwest Oncology Group. *J Clin Oncol* **11**: 873–878.

Giaccone G, Splinter TA and Debruyne C (1998) Randomized study of paclitaxel-cisplatin versus cisplatin-teniposide in patients with advanced non-small-cell lung cancer. The European Organization for Research and Treatment of Cancer Lung Cancer Cooperative Group *J Clin Oncol* **16**: 2133–2141

Gralla RJ, Casper ES, Kelsen DP, Braun DP, Dukeman ME, Martini N, Young CW, and Golbey RB (1981) Cisplatin and vindesine combination chemotherapy for advanced carcinoma of the lung: a randomized trial investigating two dosage schedules. *Ann Intern Med* **95**: 414–420

Grilli R, Andrew D, Oxman AD and Julian JA (1993) Chemotherapy for advanced non-small-cell lung cancer: how much benefit is enough? *J Clin Oncol* **11**: 1866–1872

Jaakkimainen L, Goodwin PJ, Pater J, Warde P, Murray N and Rapp E (1990) Counting the costs of chemotherapy in a National Cancer Institute of Canada randomized trial in non small-cell lung cancer. *J Clin Oncol* **8**: 1301–1309

Kawahara M, Furuse K, Kodama N, Yamamoto M, Kubota K, Takada M, Negoro S, Kusunoki Y, Matui K and Takifuji N (1991) A randomized study of cisplatin versus cisplatin plus vindesine for non-small cell lung carcinoma. *Cancer* **68**: 714–719

Klastersky J, Sculier JP, Bureau G, Libert P, Ravez P, Vandermoten G, Thiriaux J, Lecomte J, Cordier R, Dabouis G, Brohée D, Thémelin L and Mommen P for the Lung Cancer Working Party (1989) Cisplatin versus cisplatin plus etoposide in the treatment of advanced non-small-cell lung cancer. *J Clin Oncol* **7**: 1087–1092

Klastersky J, Sculier JP, Lacroix H, Dabouis G, Bureau G, Libert P, Richez M, Ravez P, Vandermoten G, Thiriaux J, Cordier R, Finet C, Berchier MC, Sergysels R, Mommen P and Paesmans M (1990) A randomized study comparing cisplatin or carboplatin with etoposide in patients with advanced non-small-cell lung cancer: European Organization for Research and Treatment of Cancer protocol 07861. *J Clin Oncol* **8**: 1556–1562

Klastersky J, Sculier JP, Ravez P, Libert P, Michel J, Vandermoten G, Rocmans P, Bonduelle Y, Mairesse M, Michiels T, Thiriaux J, Mommen P and Dalesio O and the EORTC Lung Cancer Working Party (1986) A randomized study comparing a high and a standard dose of cisplatin in combination with etoposide in the treatment of advanced non-small-cell lung carcinoma. *J Clin Oncol* **4**: 1780–1786.

Le Chevalier T, Brisgand D, Douillard JY, Pujol JL, Alberola V, Monnier A, Rivière A, Lianes P, Chomy P, Cigolari S, Gottfried M, Ruffie P, Panizo A, Gaspard MH, Ravaiolo A, Benseval M, Besson F, Martinez A, Berthaud P and Tursz T (1994) Randomized study of vinorelbine and cisplatin versus vindesine and cisplatin versus vinorelbine alone in advanced non-small-cell lung cancer: results of a European multicenter trial including 612 patients. *J Clin Oncol* **12**: 360–367

Luedke DW, Einhorn L, Omura GA, Sarma PR, Bartolucci AA, Birch R and Greco FA (1990) Randomized comparison of two combination regimens versus minimal chemotherapy in non-small cell lung cancer: a Southeastern Cancer Study Group trial. *J Clin Oncol* **8**: 886–891

Marino P, Pampallona S, Preatoni A, Cantoni A and Invernizzi F (1994) Chemotherapy vs supportive care in advanced non-small cell lung cancer. Results of a meta-analysis of the literature. *Chest* **106**: 861–865

Meert AP, Berghmans T, Branle F, Lemaitre F, Mascaux C, Rubesova E, Vermylen P, Paesmans M and Sculier JP (1999) Phase II and III studies with new drugs for non-small cell lung cancer. A systematic review of the literature with a methodology quality assessment. *Anticancer Res* **19**: 4379–4390

Mylonakis N, Tsavaris N, Bacoyiannis C, Karvounis N, Kakolyris S, Karabelis A, Beer M and Kosmidis P (1992) A randomized prospective study of cisplatin and vinblastine versus cisplatin, vinblastine and mitomycin in advanced non-small cell lung cancer. *Ann Oncol* **3**: 127–130

Paesmans M and Sculier JP (1998) Facteurs pronostiques des cancers bronchopulmonaries. In *Cancers Bronchopulmonaires*, Milleron B and Depierre A (eds) pp. 239–247. Vélizy-Villacoublay: Arnette

Paesmans M, Sculier JP, Libert P, Bureau G, Dabouis G, Thiriaux J, Michel J, Van Cutsem O, Sergysels R, Mommen P and Klastersky J for the European Lung Cancer Working Party (1995) Prognostic factors for survival in advanced non small cell lung cancer; uni and multivariate analyses including recpam analysis in 1052 patients. *J Clin Oncol* **13**: 1221–1230

Paesmans M, Sculier JP, Libert P, Bureau G, Dabouis G, Thiriaux J, Michel J, Van Cutsem O, Sergysels R, Mommen P and Klastersky J for the European Lung Cancer Working Party (1997) Response to chemotherapy has a discriminant value on the distribution of further survival for patients with advanced non small cell lung cancer: a ten years experience of the European Lung Cancer Working Party. *Eur J Cancer* **33**: 2326–2332

Robert F, Omura GA, Birch R, Krauss S and Oldham R (1984) Randomized phase III comparison of three doxorubicin-based chemotherapy regimens in advanced non-small cell lung cancer: a Southeastern Cancer Study Group trial. *J Clin Oncol* **2**: 391–395

Rosso R, Salvati F, Ardizzoni A, Curcio CG, Rubagotti A, Belli M, Castagneto B, Fusco V, Sassi M, Ferrara G, Pizza A, Pedicini T, Soresi E, Scoditti S, Cioffi R, Folco U, Monaco M, Merlano M, Rimoldi R, Tonachella R, Cruciani A, Colantuoni G, Rinaldi M, Portalone L, Bruzzi P and Santi L (1990) Etoposide versus etoposide plus high-dose cisplatin in the management of advanced non-small cell lung cancer. *Cancer* **66**: 130–134

Sculier JP, Berghmans T, Castaigne C, Lalami Y, Luce S, Sotiriou C, Vermylen P and Paesmans M (1999*a*) Best supportive care or chemotherapy for stage IV non small cell lung cancer. In *Progress and Perspectives in Lung Cancer*. Van Houtte P, Klastersky J and Rocmans P (eds) pp. 199–207. Berlin: Springer Verlag

Sculier JP, Klastersky J, Giner V, Bureau G, Thiriaux J, Dabouis G, Efremidis A, Ries F, Berchier MC, Sergysels R, Mommen P and Paesmans M for the European Lung Cancer Working Party (1994*a*) Phase II randomized trial comparing high-dose cisplatin with moderate-dose cisplatin and carboplatin in patients with advanced non-small-cell lung cancer. *J Clin Oncol* **12**: 353–359

Sculier JP, Paesmans M, Bureau G, Giner V, Lecomte J, Michel J, Berchier MC, Van Cutsem O, Küstner U, Kroll F, Sergysels R, Mommen P and Klastersky J for the European Lung Cancer Working Party (1996) A randomized trial comparing induction chemotherapy versus induction chemotherapy followed by maintenance chemotherapy in small cell lung cancer. *J Clin Oncol* **14**: 2337–2344

Sculier JP, Paesmans M, Libert P, Bureau G, Dabouis G, Thiriaux J, Michel J, Van Cutsem O, Schmerber J, Giner V, Berchier MC, Sergyselq R, Mommen P and Klastersky J for the European Lung Cancer Working Party (1994*b*) Long-term survival after chemotherapy containing platinum derivatives in patients with advanced unresectable non small cell lung cancer. *Eur J Cancer* **30A**: 1342–1347

Sculier JP, Paesmans M, Thiriaux J, Lecomte J, Bureau G, Giner V, Efremidis A, Lafitte JJ, Berchier MC, Alexopoulos CG, Zacharias C, Mommen P, Ninane V, Klastersky J and the European Lung Cancer Working Party (1998) Phase III randomized trial comparing cisplatin and carboplatin with or without

 *© 2000 Cancer Research Campaign*

ifosfamide in patients with advanced non-small-cell lung cancer. *J Clin Oncol* **16**: 1388–1396

Sculier JP, Paesmans M, Thiriaux J, Lecomte J, Bureau G, Giner V, Koumakis G, Lafitte JJ, Berchier MC, Alexopoulos CG, Zacharias C, Mommen P, Ninane V, Klastersky J and the European Lung Cancer Working Party (1999*b*) A comparison of methods of calculation for estimating carboplatin AUC with a retrospective pharmacokinetic–pharmacodynamic analysis in patients with advanced non-small cell lung cancer. *Eur J Cancer* **35**: 1314–1319

Shinkai T, Eguchi K, Sasaki Y, Tamura T, Ohe Y, Kojima A, Oshita F and Saijo N (1991) A randomized clinical trial of vindesine plus cisplatin versus mitomycin plus vindesine and cisplatin in advanced non-small cell lung cancer. *Eur J Cancer* **27**: 571–575

Shinkai T, Saijo N, Eguchi K, Sasaki Y, Tominaga K, Sakurai M, Suga J, Miyoaka H, Sano T, Keicho N, Takahashi H, Ishihara J, Tamura T, Hoshi A and Jett JR (1986) Cisplatin and vindesine combination chemotherapy for non-small cell lung cancer: a randomized trial comparing two dosages of cisplatin. *Jpn J Cancer Res* **77**: 782–789

Souquet PJ, Chauvin F, Boissel JP, Cellerino R, Comier Y, Ganz A, Kaasa S, Pater JL, Quoix E, Rapp E, Tumarello D, Williams J, Woods BL and Bernard JP (1993) Polychemotherapy in advanced non-small-cell lung cancer: a meta-analysis. *Lancet* **342**: 19–21

Thongprasert S, Sanguanmitra P, Juthapan W and Clinch J (1999) Relationship between quality of life and clinical outcomes in advanced non-small cell lung cancer: best supportive care (BSC) versus BSC plus chemotherapy. *Lung Cancer* **24**: 17–24

Weick JK, Crowley J, Natale RB, Hom BL, Rivkin S, Coltman CA, Taylor SA and Livingston RB (1991) A randomized trial of five cisplatin-containing treatments in patients with metastatic non-small-cell lung cancer: a Southwest Oncology Group Study. *J Clin Oncol* **9**: 1157–1162

Wozniak AJ, Crowley JJ, Balcerzak SP, Weiss GR, Spiridonidis CH, Baker LH, Albain KS, Kelly K, Taylor SA, Gandara DR and Livingston RB (1998) Randomized trial comparing cisplatin with cisplatin plus vinorelbine in the treatment of advanced non-small-cell lung cancer: a Southwest Oncology Group study. *J Clin Oncol* **16**: 2459–2465

### APPENDIX

### European Lung Cancer Working Party participating institutions, with investigators:

Institut Jules Bordet, Brussels, Belgium
JP Sculier
T Berghmans
P Van Houtte
J Klastersky
M Paesmans
P Mommen

Hôpital Saint-Pierre, Brussels, Belgium
V Ninane
T Bosschaerts
R Sergysels

Hôpital Albert Calmette, Lille, France
JJ Lafitte

CHV de Charleroi, Charleroi, Belgium
J Thiriaux
J Lecomte
A Wattiez

Evangelismos General Hospital, Athens, Greece
CG Alexopoulos
M Vaslamatzis

Klinika Radiotherapie a Onkologie, Kosice, Slovakia
J Baumöhl

Hôpital Brugmann, Brussels, Belgium
A Drowart
T Prigogine

Hellenic Cancer Institute, Athens, Greece
A Efremidis
G Koumakis

CH de Douai, Douai, France
MC Florin
E Maetz

CHI de Montfermeil, Montfermeil, France
C Zacharias

Hôpital Ambroise Paré, Mons, Belgium
P Recloux

Hospital de Sagunto, Valencia, Spain
V Giner

CH de Roubaix, Roubaix, France
F Kroll
F Steenhouwer
A Strecker

Hôpital d'Hayange, Hayange, France
MC Berchier

Clinique Saint-Luc, Namur, Belgium
O Van Cutsem
M Mairesse

Clinique Louis Caty, Baudour, Belgium
V Richard
D Diana

CHU A Vésale, Montigny-le-Tilleul, Belgium
D Brohée

Hôpital de Warquignies, Boussu, Belgium
P Libert
M Richez

CH Peltzer-La Tourelle, Verviers, Belgium
JL Corhay

CHG de Tourcoing, Tourcoing, France
X Ficheroulle

Hôpital de la Madeleine, Ath, Belgium
P Ravez

Hôpital de Braine-l'Alleud, Braine-l'Alleud, Belgium
C Finet

Cabinet Médical de Dampierre, Anzin, France
B Stach
JP Roux

Clinique de La Louvière, Lille, France
F Fortin
JM Dernis
JM Grosbois

IMC Mutualités Socialistes, Tournai, Belgium
A Tagnon
G Nuttin

Centre Hospitalier Dr Schaffner, Lens, France
J Amourette

Hôpital Duchenne, Boulogne sur Mer, France
JL Crépin

Cabinet de Pneumologie, Tourcoing, France
Y Watrigant

© 2000 Cancer Research Campaign
